# Stability testing of dried *Plasmodium falciparum* positive quality control samples for malaria rapid diagnostic tests in Liberia and Benin

**DOI:** 10.1186/s12936-020-03364-9

**Published:** 2020-08-12

**Authors:** Saliou Ramani, Henry T. Kohar, Oliver Pratt, Yves Eric Denon, Christie M. Reed, Peter Thomas, Suzanne E. Powell, Michael Aidoo

**Affiliations:** 1 ARM3 Project, Cotonou, Benin; 2 National Malaria Control Programme, Monrovia, Liberia; 3 National Malaria Control Programme, Cotonou, Benin; 4 US President’s Malaria Initiative, Division of Parasitic Diseases and Malaria, Center for Global Health, Centers for Disease Control and Prevention, Monrovia, Liberia; 5 US President’s Malaria Initiative, Division of Parasitic Diseases and Malaria, Center for Global Health, Centers for Disease Control and Prevention, Cotonou, Benin; 6 US President’s Malaria Initiative, Division of Parasitic Diseases and Malaria, Center for Global Health, Centers for Disease Control and Prevention, 1600 Clifton Road, Atlanta, GA 30333 USA

**Keywords:** Malaria, Rapid test, Quality control, Dried tube specimen

## Abstract

**Background:**

Malaria rapid diagnostic tests (RDTs) are largely responsible for the gains made in the proportion of malaria cases confirmed with a parasitological test. However, quality assurance programs to support their use remain a challenge. A dried tube specimen (DTS) method was developed that showed potential for use as a stable source of quality control (QC) sample for RDTs and for use in external quality assessments or proficiency testing (PT). DTS was further assessed with focus on sample stability under field settings in Benin and Liberia.

**Methods:**

DTS were prepared using *Plasmodium falciparum* 3D7 or W2 strains at concentrations of 1000, 500 or 0 parasites/µL and tested for baseline reactivity at the Centers for Disease Control and Prevention, Atlanta before shipping. In Benin and Liberia, DTS were stored under refrigeration in a reference laboratory (RL) or in health centres under ambient temperatures. Seven rounds of testing were performed at 4-week intervals during which DTS were tested on RDTs stored at the RL or at health centres. Observed DTS reactivity at the RL and health centres were compared to expected reactivity to determine DTS stability. DTS were also assembled into a PT panel and tested by health facility staff at the mid and end time-points of the study. Daily maximum and minimum storage temperatures for RDTs and DTS were recorded.

**Results:**

In Benin, DTS, irrespective of storage conditions, produced the expected reactivity at all time points. However, evidence of degradation was observed at weeks 20 and 24 for DTS stored at ambient temperatures at the health centres and not those stored under refrigeration at the RL. In Liberia, sample degradation was observed starting at week 8 especially among DTS stored at the health facilities. The degradation was associated with prolonged storage of DTS under ambient temperature prior to study commencement and less than optimal storage temperatures at the RL. Use of DTS in a PT enabled identification of health worker errors in performing the tests.

**Conclusion:**

DTS is a feasible tool for use as QC material and for PT under field conditions. Long-term (> 5 months) storage of DTS requires refrigeration.

## Background

There has been a substantial increase in the use of malaria rapid diagnostic tests (RDTs) since the World Health Organization (WHO) recommended in 2010 that all suspected malaria cases be parasitological confirmed with a quality-assured test and anti-malarial treatment provided only to test positive cases [1]. Diagnostic testing of suspected malaria cases in sub-Saharan Africa has since increased from 36% in 2010 to 82% in 2017, an increase primarily due to the uptake of RDTs which accounted for 40% and 75% of all testing in 2010 and 2018, respectively [2]. RDTs are increasingly being used outside the laboratory setting by community health workers and volunteers operating in small communities [3–6]. This increase in RDT use outside the laboratory and by non-laboratory trained health workers requires the establishment of a robust quality assurance (QA) system, a key requirement of the WHO case management guidelines, to guarantee the delivery of accurate test results for appropriate patient management.

Despite the increased use of RDTs outside the laboratory, QA systems to support such use are inadequate. The WHO conducts an annual RDT product-testing programme that monitors the performance of RDTs as provided by manufacturers [7]. In addition, the WHO provides a pre-procurement lot testing service for large global institutions such as the Global Fund and the US President’s Malaria Initiative. However, these efforts are inadequate to address the quality of tests and at the point-of-care in the field. Malaria RDT products are not supplied with control samples and quality checks at the point-of-care are lacking. Recent efforts to develop quality control (QC) material has focused on recombinant antigens [8] that has been shown to improve health worker adherence to treatment guidelines as a result of trust in RDTs. However, a product universally reactive similar to native antigen on all or most RDTs is still lacking.

A dried tube sample (DTS) method that preserves target parasite antigens in their native forms in blood can be used to generate QC samples for RDT QA [9]. In order to further assess the feasibility of using DTS to support QA in the field, especially its use as QC and proficiency testing samples, three field studies were conducted in Ethiopia, Benin and Liberia under country-specific conditions. The primary objective of the field assessments was to determine the stability of DTS under ambient temperature conditions in sub-Saharan Africa compared to refrigeration. In addition, the suitability of DTS for use as samples in a proficiency-testing programme was assessed. The preferred DTS product would be stable at ambient temperatures to enable storage at testing sites and used as needed. In the Oromia region of Ethiopia where average maximum temperatures range from 19 to 24 °C, DTS was found to be stable for the 6-month evaluation period when stored at ambient temperatures at health centres [10]. In order to further assess DTS stability under temperature conditions typically found in tropical sub-Saharan Africa, field assessments similar to that conducted in Ethiopia were conducted in Liberia and Benin both with average maximum temperature range from about 28 °C to 32 °C (https://www.worldweatheronline.com).

## Methods

### DTS preparation

DTS were prepared as previously described [9]. Briefly, culture-adapted *Plasmodium falciparum* parasites were grown in culture, harvested and diluted to desired parasite densities in washed (in incomplete medium; RPMI 1640, Gibco™ Thermo Fisher, Grand Island, NY, USA) parasite negative blood at similar haematocrit. Parasite dilutions of 1000, 500 or 0 parasites/µL of blood were produced for this study. The diluted parasite preparations were then tested at the Center for Disease Control (CDC) Malaria Branch Laboratory on malaria RDT brands from three different manufacturers to determine baseline reactivity. After confirmation of reactivity, the diluted parasites were distributed in 50-µL aliquots in Sarstedt^®^ Type I micro tubes (Sarstedt Inc., Newton, NC, USA) and with the caps open air-dried overnight in a biosafety cabinet. To check that baseline reactivity was not affected by drying, 1 vial each of DTS at 1000, 500 and 0 parasites/µL were rehydrated using a solution of PBS-Tween20 (Sigma Aldrich, St. Louis, MO, USA) and tested on the same RDTs brands as prior to drying.

DTS containing 1000, 500 and 0 parasites/µL were prepared from culture-derived *P. falciparum* in enough quantity for training of staff and the main study and were stored under refrigeration until shipped to country. DTS were tested on three RDT brands at the Malaria Laboratory, CDC, Atlanta, before shipping at ambient temperature to both countries. In country, replicate DTS were stored under refrigeration (~ 4 °C) at a reference laboratory and at ambient temperature at the health centres.

DTS for Liberia were prepared using culture-derived *P. falciparum* strain W2 in December 2011 with an expected start date of first quarter of 2012, however, study start date was postponed until the third quarter of 2013. DTS were stored in Liberia at ambient temperature during this delay. DTS for Benin were prepared using culture-derived *P. falciparum* strain 3D7 in May 2014 with an expected study commencement date in July 2014. Unlike in Liberia, no delays in study start date were experienced.

The lower parasite density threshold of 500 parasites/µL was used although currently, the WHO estimates RDT performance using a lower parasite density threshold of 200 parasites/µL. The 200 parasites/µL threshold has been shown in seven rounds of RDT product testing to be sufficient in distinguishing high-performing from low-performing tests [7]. In this study, the DTS lower limit was set at a slightly higher level of 500 parasites/µL in order to remove or minimize test-specific non-reactivity as opposed to non-reactivity due to deterioration of the DTS.

### Rapid diagnostic tests

RDT brands commonly used in public sector facilities in the respective countries were procured directly from the manufacturers for the study. *P. falciparum* (HRP2)-specific SD Bioline RDTs (SD Bioline Malaria Ag Pf, catalogue # 05FK50, Standard Diagnostics, Seoul, South Korea), were procured and shipped directly to Benin by the manufacturer. First Response Malaria Antigen Pf (HRP2) RDTs (First Response Malaria Antigen Pf, catalogue # I13FRC, Premier Medical Corporation, Nani Daman, India), obtained directly from the manufacturer and shipped to Liberia from CDC, Atlanta. All RDTs were performed according to manufacturer instructions. Reference laboratory RDTs were stored under refrigeration in Benin but in an air-conditioned room in Liberia. Health centre RDTs were stored at ambient temperature in both countries.

### Facilities

In both countries, one reference laboratory for optimal storage of tests and DTS and two public health centres were chosen as sites for the study. The health centres in both countries provided care to mainly urban populations and provided a similar level of care. In Benin, the Centre for Entomological Research (CREC) functioned as a reference laboratory, while St. Michel Health Centre, Cotonou 5, and Ayelawadje Health Centre, Cotonou 2–3 were the clinical sites. In Liberia, the facilities chosen for the study were the National Drug Quality Control Laboratory, Monrovia, as the reference laboratory (RL), and Slipway Community Clinic (SLP) and Star of the Sea Health Centre (SoS), both in Monrovia as the testing sites. In both countries, facilities were chosen so that all facilities could be visited and testing conducted within 8 h of sample rehydration. Approximately 1 week prior to study commencement, study staff visited all sites to introduce the study to the facility administrators and laboratory staff and to supply RDTs, DTS, temperature monitors and timers.

### Training

Study coordinators in the each of the two countries were trained on standard procedures for rehydration and testing DTS and on procedures to follow on each testing day. Facility staff received training on testing DTS since they were expected to test a proficiency panel at study weeks 12 and 24. Bench aids for DTS rehydration and RDT testing were provided to all laboratory staff in all facilities.

### Testing scheme

#### DTS testing

The study commenced with week 0 testing. Each DTS was tested on duplicate RDTs and results were considered satisfactory if both RDTs produced the correct result. Testing was done every 4 weeks for a period of 6 months. At weeks 12 and 24, health centre staff who routinely perform RDTs and who received prior training on the use of the DTS were asked to take part in testing of a 4-sample proficiency panel in Liberia and a 3-sample proficiency panel in Benin.

On the day of testing, DTS stored at the RL under refrigeration were rehydrated and tested on duplicate RDTs stored under refrigeration in Benin and at ambient temperature in Liberia. DTS and RDTs that were refrigerated were brought to ambient temperature before testing. This testing of RL RDT and DTS was considered control testing for health centre RDT and DTS. The same set of rehydrated DTS and RDTs from the RL were then transported to the two health centres where the RL DTS were tested in duplicate on RDTs stored at ambient temperatures at the health centre. In addition, DTS stored at ambient temperature at the health centres were rehydrated and tested in duplicate on the RL-stored RDTs and RDTs stored at the health centre. This process (Additional file 1) was followed on each testing day every 4 weeks for 6 months from the first time point (week 0) for a total of seven time points.

#### Proficiency testing (PT)

At weeks 12 and 24, health centre staff were challenged with a proficiency panel of reconstituted specimens labelled with random sample identification numbers. In Benin, the PT panel consisted of one sample each of 1000, 500 and 0 parasites/µL while in Liberia, the panel consisted of four DTS samples: one sample at 1000 parasites/µL, two samples at 500 parasites/µL and one negative (0 parasites/µL). In both countries, the DTS samples in the proficiency panel were stored under refrigeration in the RL and reactivity known only to the study coordinator. Prior to arriving at the health centre, the study coordinator rehydrated the samples in the PT panel, tested them on RL-stored RDTs to confirm their expected reactivity and labelled them with a random sample identity number. At the health centres, the facility staff were provided with rehydrated proficiency panel and RL-stored RDTs for testing. During testing of the PT panel by health centre staff, the study coordinator observed the technician and recorded correct and incorrect testing procedures using a checklist of nine critical steps in the RDT testing procedure (Additional file 2). In addition, a pass/fail form (Additional file 3) was completed by the study coordinator to indicate whether the technician read and recorded the correct sample reactivity. Errors were noted irrespective of the number of times it was made for each of the samples in the PT panel. Feedback regarding health worker performance was provided to health centre staff and corrections provided when needed.

#### Temperature monitoring

In order to determine the temperatures that RDTs and DTS were exposed to at each facility during the study period, maximum and minimum storage temperatures were recorded every 24 h by use of a Big Digit Indoor/Outdoor maximum and minimum thermometer (Extech Instruments, New Hampshire, USA). Over weekends and public holidays when staff were unavailable to record temperatures, maximum and minimum temperatures for the duration of time from the last measured temperatures were recorded. The maximum and minimum thermometer operates by measuring temperatures continuously and when prompted by an integrated button will display the lowest and highest temperature recorded since the last reset. After temperatures are recorded, the thermometer is reset to begin a new cycle of maximum and minimum temperature measurements. For this activity, the thermometer was reset daily after maximum and minimum temperatures were recorded. The maximum and minimum thermometer does not estimate the period over which a particular temperature measurement has been sustained.

## Results

### Benin

#### Reference laboratory DTS testing on reference laboratory and health centre RDTs

Considering replicate test (2 each of 1000, 500 or 0 parasites/µL) of each DTS as a single test, all combinations of RL-stored DTS and RDTs for all facilities were concordant (100%) with the expected results for all time points from week 0 to 24. (Table 1A, top two panels) reflecting stability of DTS stored at 4 °C over the 7-month study period.

**Table 1:**
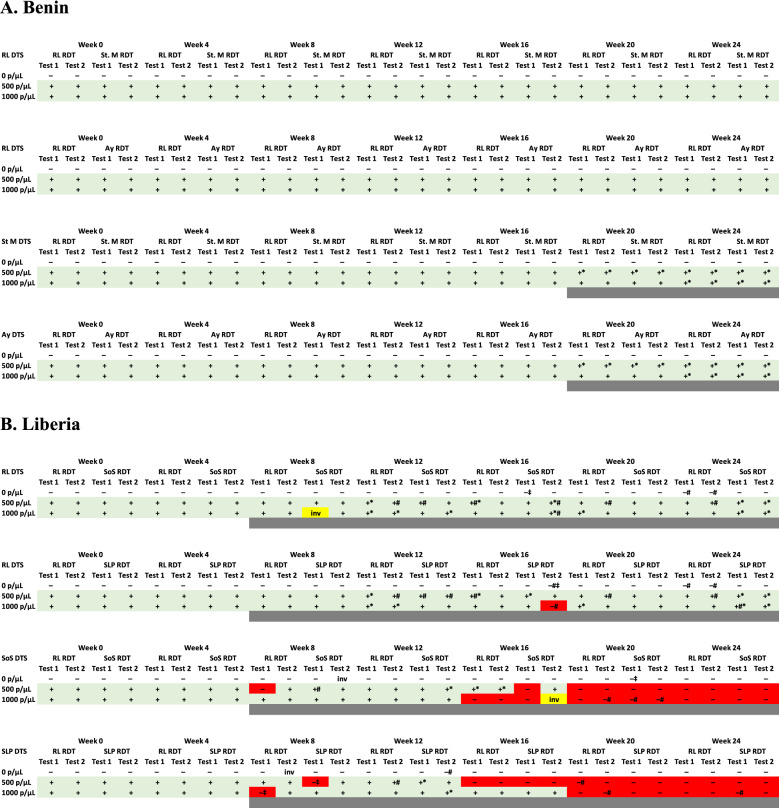
Reference laboratory and health centre-stored DTS reactivity on reference laboratory and health centre-stored RDTs for Benin and Liberia

Results of RL DTS on RL RDTs are repeated in the top 2 panels of each table for ease of comparison with RL DTS on health centre RDTs

^#^Invalid tests repeated; *inv* invalid test not repeated (yellow highlight); * faint test line; ^‡^ Faint control line; grey highlight below each panel denotes time points during which incomplete rehydration was observed; red highlight denotes false negative DTS results. *Ay* Ayelawadje health centre; *St. M* St. Michel health centre; *SoS* Star of the Sea health centre; *SLP* Slipway Community Clinic

#### Health centre DTS testing on reference laboratory and health centre RDTs

Similar to RL DTS testing on RL and health centre RDTs, reactivity of health centre DTS (from both health centres) on RL and health centre RDTs were 100% concordant for all time points. However, despite 100% concordance of health centre-stored DTS with expected sample reactivity, at weeks 20 and 24, lower band intensities were observed for 500 parasites/µL DTS stored at both St Michel and Ayelawadje health centres. In addition, at week 24, health centre-stored DTS at 1000 parasites/µL from both health centres also registered as low intensity bands on all RDTs. These low band intensities (1 + compared to 4 + on time point equivalent RL DTS as measured by band intensity template) were observed when either RL or health centre-stored RDTs were used although the same RDTs showed expected band intensities when RL DTS were tested. This result suggested the low band intensities were due to the DTS and not the RDT. Indeed, at weeks 20 (for 500 parasites/µL DTS) and 24 (for both 500 and 1000 parasites/µL DTS) at both health centres, rehydration of health centre-stored DTS was observed to be incomplete with a gel-like mass at the base of the tube that, when applied to the RDT, did not flow efficiently on the test strip.

#### Proficiency testing of health centre staff

At week 12, three technicians at Ayelawadje health centre were challenged with a 3-sample PT panel consisting of one each of DTS at 1000, 500 and 0 parasites/µL, randomly labelled with a generic sample number. One technician performed all the 9 observed critical steps correctly. The second technician made 3 errors in the 9 steps including collecting and applying an incorrect volume of blood and adding 3 instead of 4 drops of buffer to the test. The third technician made errors in 5 of the 9 steps including collecting and applying more sample volume, applying 2 instead of 4 drops of buffer, not setting a timer and reading the result at 7 instead of 15 min. The number of times an error was made for each of the samples in the panel was not recorded. Despite these errors, the sample reactivity was unaffected and the technicians interpreted test results correctly.

At St Michel, four technicians were challenged with the PT panel. All four technicians made at least one error. One technician made only one mistake applying an incorrect (less than required by test) volume of blood. The second technician added 3 instead of 4 drops of buffer, set the wrong time and read the test in that time (5 instead of 15 min). The third technician applied an incorrect volume of blood and set an incorrect time to read the test. Despite these errors, the test reactivity was as expected and all three technicians scored the PT samples correctly. The fourth technician re-used a sample collection pipette for another sample in the PT panel, did not set a timer, read the test in 7 min and scored the negative sample incorrectly as positive. Although the sample was negative, the test appeared as positive due to cross-contamination caused by re-use of the blood-collecting pipette from a sample that was positive.

At week 24, PT was done by two health workers in each health centre none of whom took part in the week 12 testing. At St Michel, the health workers performed all procedural steps correctly. However, at the Ayelawadje health centre, the two health workers made 4 and 5 procedural errors. The technician who made 4 errors collected and applied incorrect blood volume, did not set a time and read the test in less time than required by the test (time not recorded in result sheet). The technician who made 5 errors did not set a time (and therefore no correct time set), read the test in less time than required by the test (time not recorded in result sheet) and interpreted the result incorrectly. For the latter a positive sample was scored as negative and a negative sample was scored as invalid.

#### Storage temperature monitoring

**Fig. 1:**
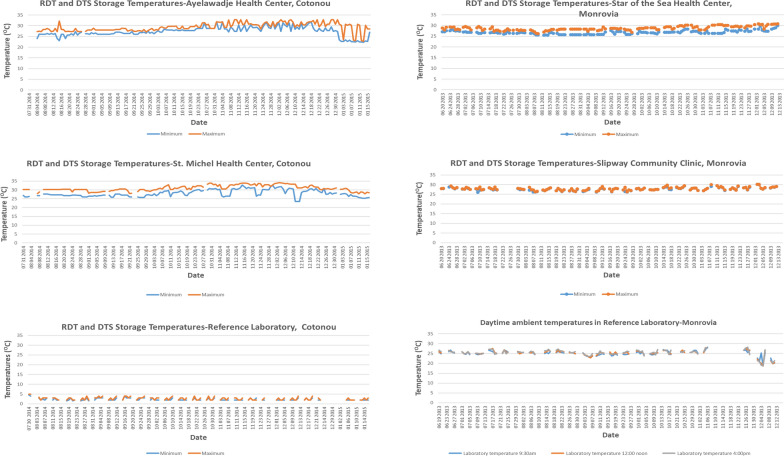
Temperature monitoring charts

Maximum and minimum RDT and DTS storage temperature measurements for the RL and the health centres are shown in Fig. 1 (left panel). At Ayelawadje health centre, minimum daily temperatures rose steadily over the 6 months from an average of about 26.8 °C to 30.4 °C. Minimum temperatures at St Michel health centre were on average 27.4 °C in the first 3 months rising to 28.8 ^O^C in the last 3 months while average maximum temperatures rose from 28.8 ^O^C to 31.7 ^O^C during the same period. RDTs and DTS at the RL were stored in a laboratory refrigerator with measured temperatures of between 2.1 and 2.4 °C (within reading error range of the thermometer).

### Liberia

#### Reference laboratory DTS testing on reference laboratory and health centre RDTs

In Liberia RL DTS reactivity on RL RDTs were concordant with the expected results (100%) for all time points. However, starting from week 12 up to week 24, several tests of the RL DTS gave invalid results (no control band) that needed to be repeated or showed low band intensities (Table 1B, top two panels). Observations of these invalid and low band intensity results coincided with observations by study staff of incomplete DTS sample rehydration as described above for Benin.

RL DTS produced expected results on all health centre RDTs except for two time points. At week 8, a RL DTS at 1000 p/µL produced an invalid test on one of the replicates of the SoS RDTs. That test was not repeated as required by study protocol. In addition, at week 16, a RL DTS at 1000 p/µL produced an invalid test on one replicate of SLP RDTs. This test was repeated as required by the study protocol and returned a false negative result (Table 1B, top two panels).

#### Health centre DTS on reference laboratory and health centre RDTs

Health centre DTS from both facilities were concordant with expected results (100%) up to week 4 when tested on RL or health centre RDTs (Table 1B, lower two panels). However, similar to observation of the RL DTS but in this case, starting from week 8, study staff begun to notice and record incomplete sample rehydration. Results of tests performed with incompletely rehydrated DTS samples ranged from expected test results with faint bands (for positive samples), false negative or invalid test results (Table 1B, lower two panels). Invalid tests repeated once as required by the study protocol produced expected results in weeks 8 and 12 albeit with faint bands. However, from week 16 to week 24, replicates of positive DTS samples that did not fully rehydrate and initially produced invalid results were false negative on the second test (red shaded blocks, Table 1B, lower two panels).

#### Proficiency testing of health centre staff

At week 12, one technician at SLP and one of the two technicians at SoS were challenged with a PT panel consisting of four randomly labelled DTS: 1000 (1), 500 (2) and 0 (1) parasites/µL, all of which were scored correctly. However, at SLP, the technician made 6 errors in the 9-step checklist for at least one of the 4 tests. The technician collected and applied incorrect sample volume to the test, dispensed incorrect number of buffer drops (number of drops not indicated) for some tests and did not set a timer or make note of time to read test. In addition, all test results were read at a time earlier than specified by the manufacturer. The technician from SoS made 2 errors in the 9-step checklist; collecting and applying more blood sample volumes than required by test for at least 3 of the tests in the panel. Despite these errors, results appeared as expected and were correctly read by the technician.

The same laboratory technicians from week 12 performed PT at week 24. At SLP, all procedural steps were performed correctly except for incorrect volume (more than required by test) of blood collected and applied to two of the four tests in the panel. However, at SoS, errors in collection of appropriate volume into pipette and application on test as well as incubations times not being recorded remained a problem. In all cases, test reactivity was as expected and result interpretations were correct.

#### Storage temperature monitoring

Storage temperatures for DTS and RDTs at the health facilities showed that these supplies were not exposed to high temperatures for prolonged periods (Fig. 1, right panel). At the RL, only ambient temperatures were recorded and therefore were relevant only for RDTs that were stored on laboratory shelves. In addition, the maximum-minimum thermometer provided for the study was not used. Instead, the laboratory relied on its own established temperature monitoring system which required recording ambient temperatures at 09:00, 12.00 and 16:00 h each work day. On average recorded temperature within the laboratory measured only during the workday was about 25.5^O^C and ranged from 23.4 ^O^C to 28.2 ^O^C. However, it became known after the study that electrical power to the laboratory, including the refrigerator, was completely switched off at the end of each workday. These storage temperatures and temperature monitoring deviated from the study protocol. At SoS and SLP, recorded maximum daily temperatures were below 30 °C up to week 20 after which temperatures rose slightly but above 30 °C for a few days but never above 31 °C (Fig. 1, right panel).

## Discussion

During the 6-month assessment period, the utility of DTS as a viable field deployable tool for malaria RDT QC was assessed. The assessment was designed to determine the stability of DTS at ambient temperatures typical of sub-Saharan Africa where most RDTs are used in order to inform how the DTS will be used within a programme setting. The two conditions considered were DTS kept at health centres and used as needed by health centre staff or DTS stored under refrigeration in a RL and sent to health centres at pre-determined intervals thus limiting storage at the health centre and exposure to high ambient temperatures.

In Benin, DTS stored at 4 °C at the RL remained stable over the study period without effects on rehydration or the resulting band intensity on tests similar to observations in a similar study in Ethiopia [10]. That result also reflects previous reports and observations that DTS when stored at 4 °C are stable for over 2 years [9] and up to 4 years, (unpublished). However, when stored at ambient temperature in health facilities, DTS was stable for up to the week-16 time point after which sample degradation was observed manifest as incomplete rehydration and formation of a gel-like mass.

In Liberia, DTS from both the health centres and the RL were unstable. DTS for Liberia differed from those in Benin in two ways. First, they were stored for an extended period due to the delay of over 12 months associated with implementation noted above. During this interval, the DTS were stored at ambient temperature of approximately 25 °C. Despite this delay, testing of DTS in May 2013 immediately before study commencement showed normal rehydration, reactivity and test band intensities comparable to that immediately after DTS preparation in 2011. Second, it became known after study completion that electricity to the reference laboratory in Liberia was in fact not continuous and that the on-site power generation units were turned off at the end of each workday and on weekends. As this was not discovered until much later, the study relied on the laboratory’s system of recording ambient temperatures manually instead of the continuous temperature monitoring. As a result of the power interruptions, the DTS were likely exposed to temperatures > 4 °C for an unknown period during the study. This was a deviation from the study protocol. However, the results from Liberia are not discarded but rather included in the study report as they provide additional information on DTS storage temperatures that have adverse effects on stability. It appears that, together, prolonged storage and exposure of DTS to elevated temperatures likely affected DTS integrity in Liberia resulting in incomplete rehydration that in turn affected reactivity. Despite the less than optimal storage conditions at the RL and the age of DTS, the results of RL-stored DTS compared to the health centre-stored DTS (Table 1B) suggest degradation was slower at the RL where temperatures were lower overall than ambient temperatures at the health centres (Fig. 1, right panel).

Based on the incomplete rehydration of DTS and the resulting low band intensities observed at weeks 20 and 24 in Benin and the observed DTS deterioration in Liberia it appears that long-term storage of DTS should be under refrigeration (~ 4 °C). It is unclear what length of time at a given ambient temperature in sub-Saharan Africa DTS stability is adversely affected. However, even at the measured temperatures (maximum daily temperatures of between 27^O^C and 32^O^C) DTS was stable up to at least 4 months in Benin. In Ethiopia where temperatures were likely slightly lower than in Benin and Liberia, DTS were stable throughout the 6-month study period [10]. These observations help to provide guidance on how a DTS-based QC system and PT programme could be implemented with regard to timing for sample preparation and shipment as well as recommendations to facilities on expected shelf life of the QC samples. With stability up to about 4 months at ambient temperatures of approximately 30^O^C, this method is suitable for use in sub-Saharan Africa without the need for cold chain. However, it is important that prolonged storage beyond 4 months at such temperatures be minimized. As a comparison, HIV programmes using the DTS methodology for PT ship DTS out to health facilities requiring testing and reporting of results within one month [11]. A similar approach for a DTS-based malaria RDT PT is envisaged.

In Benin, the observed low band intensities at weeks 20 and 24 were likely caused by incomplete rehydration of health centre-stored DTS which led to sub-optimal sample lysis and antigen release. With storage temperatures being the only obvious difference in the handling of RL and health centre-stored DTS, the likely explanation is that exposure of DTS to heat for prolonged periods compromised sample integrity. Invalid RDT results may also be due to degradation of RDTs. However, post-study investigation of high invalid RDT result rates in Liberia using freshly prepared DTS on study RDTs showed the study RDTs were performing as expected (Additional file 4). Humidity could also play a role in sample degradation; however, humidity conditions were not measured during the study. These results suggest that incomplete DTS rehydration is an indication of compromised sample integrity and such DTS should not be used for QC or PT.

In addition to use as QC samples, DTS was useful in checking the competency of health workers in performing critical procedural steps in RDT testing. With the knowledge of sample reactivity, the study coordinators were able to identify and correct errors that could lead to misclassification of patient test results. All technicians in the health centres reported receiving prior RDT training and RDT was the primary means of malaria diagnosis in these facilities. However, some errors made by technicians appeared to be random and not consistent, suggesting inadequate training and lack of understanding of the tests. In one case, re-use of a sample collection device previously used on a positive DTS sample on a subsequent negative DTS sample led to contamination of the negative sample resulting in a false positive sample reactivity. Without knowledge of sample reactivity as in this PT panel or routine periodic observation of technique during on-site supervision, this type of error would have been difficult to detect in practice and remedy through training. Therefore, a PT programme based on DTS can be used to monitor and improve health worker use of malaria RDTs and could provide an added level of monitoring of health worker performance of RDTs. Precedence for this has been reported for HIV RDT PT [11, 12]. Although the study did not aim to specifically assess consequences of operational errors on test reactivity, the data suggest certain deviations from manufacturer instructions for blood and buffer volumes as well as incubation time as observed in this study do not affect sample reactivity. A systematic study of procedural errors is needed to better understand how they affect test results.

While a manufactured quality control reagent, preferably one that comes with the kit will be ideal, such materials are not yet available. In the meantime, a manufactured product, such as the recombinant antigen [8] or the DTS as described here, could be viable alternatives. The recombinant antigen has the advantage of being manufactured and, therefore, more consistent over time. However, it needs to match the product to be tested. The DTS on the other hand, while not manufactured contains native antigen and provides the flexibility of varying content to suit specific situations.

## Conclusion

The DTS method as developed [9, 10] and described here is a simple and stable source of sample for training, quality control and proficiency testing in support of the increasingly important role of malaria RDTs in malaria case management. DTS can be easily developed using either malaria-positive patient blood or in vitro culture-derived parasites. The method provides the convenience of using actual parasite material, the flexibility of varying parasite density and content to suite multiple needs in the field and stability under refrigeration for over 2 years or approximately 4 months at ambient temperature in tropical sub-Saharan Africa.

## Supplementary information

**Additional file 1.** Testing scheme for reference laboratory and health centre DTS and RDTs.

**Additional file 2.** Checklist for observing health worker performance of DTS proficiency tests.

**Additional file 3.** Proficiency testing reporting form.

**Additional file 4.** Liberia DTS study -DTS and RDT control Testing using new DTS.

## Data Availability

All data from this study are available on reasonable request.

## Abbreviations

DTS: Dried tube specimen
RL: Reference laboratory
SoS: Star of the Sea Health Centre, Liberia
SLP: Slipway Community Clinic, Liberia
PT: Proficiency testing
