# Supplementary material for: Stability testing of dried Plasmodium falciparum positive quality control samples for malaria rapid diagnostic tests in Liberia and Benin
Source: Malar J. 2020 Aug 12;19:288. doi: 10.1186/s12936-020-03364-9 (PMC7424989; doi:10.1186/s12936-020-03364-9)
Supplement: Supplementary file 1 — Additional file 1. Testing scheme for reference laboratory and health centre DTS and RDTs. [file 12936_2020_3364_MOESM1_ESM.docx]

**Additional file 1**

**Testing scheme for reference laboratory and health centre DTS and RDTs**

|  | | | |
| --- | --- | --- | --- |
| **Reference Lab DTS** | **Reference Lab RDT** | **Health Centre 1 RDT** | **Health Centre 2 RDT** |
| **1,000** | **X** | **X** | **X** |
| **500** | **X** | **X** | **X** |
| **0** | **X** | **X** | **X** |
|  |  |  |  |
|  |  |  |  |
| **Health Centre 1 DTS** | **Reference Lab RDT** | **Health Centre 1 RDT** | **Health Centre 2 RDT** |
| **1,000** | **X** | **X** |  |
| **500** | **X** | **X** |  |
| **0** | **X** | **X** |  |
|  |  |  |  |
|  |  |  |  |
| **Health Centre 2 DTS** | **Reference Lab RDT** | **Health Centre 1 RDT** | **Health Centre 2 RDT** |
| **1,000** | **X** |  | **X** |
| **500** | **X** |  | **X** |
| **0** | **X** |  | **X** |

## *X indicates testing done*
