# Supplementary material for: Stability testing of dried Plasmodium falciparum positive quality control samples for malaria rapid diagnostic tests in Liberia and Benin
Source: Malar J. 2020 Aug 12;19:288. doi: 10.1186/s12936-020-03364-9 (PMC7424989; doi:10.1186/s12936-020-03364-9)
Supplement: Supplementary file 2 — Additional file 2. Checklist for observing health worker performance of DTS proficiency tests. [file 12936_2020_3364_MOESM2_ESM.docx]

**Additional file 2**

**Checklist for observing health worker performance of DTS proficiency tests**

|  | **Yes = 1** | **No = 2** |
| --- | --- | --- |
| **Correct volume of blood collected** |  |  |
| **Correct volume of blood applied** |  |  |
| **Blood applied to sample well** |  |  |
| **Correct number of buffer drops dispensed** |  |  |
| **Buffer dispensed into buffer well** |  |  |
| **Timer set for incubation** |  |  |
| **Correct time set for incubation** |  |  |
| **Results read at time specified by test** |  |  |
| **Results interpreted correctly** |  |  |
