# Supplementary material for: Stability testing of dried Plasmodium falciparum positive quality control samples for malaria rapid diagnostic tests in Liberia and Benin
Source: Malar J. 2020 Aug 12;19:288. doi: 10.1186/s12936-020-03364-9 (PMC7424989; doi:10.1186/s12936-020-03364-9)
Supplement: Supplementary file 3 — Additional file 3. Proficiency testing reporting form. [file 12936_2020_3364_MOESM3_ESM.docx]

**Additional file 3**

**Proficiency testing reporting form**

Health Facility Name (or Code) __________________________________________________________

Laboratory Technician__________________________________________________________________

Date________________________________________________________________________________

| **PT Sample ID** | **RDT Brand (Product #)** | **RDT Result** | **RDT PT Report**  ***For QA Officer Use only*** | **Comments** |
| --- | --- | --- | --- | --- |
|  |  | ❑Positive  ❑Negative  ❑Invalid | ❑ Pass  ❑ Fail  ❑ ND |  |
|  |  | ❑Positive  ❑Negative  ❑Invalid | ❑ Pass  ❑ Fail  ❑ ND |  |
|  |  | ❑Positive  ❑Negative  ❑Invalid | ❑ Pass  ❑ Fail  ❑ ND |  |
|  |  | ❑Positive  ❑Negative  ❑Invalid | ❑ Pass  ❑ Fail  ❑ ND |  |
|  |  | ❑Positive  ❑Negative  ❑Invalid | ❑ Pass  ❑ Fail  ❑ ND |  |
