# Supplementary material for: Stability testing of dried Plasmodium falciparum positive quality control samples for malaria rapid diagnostic tests in Liberia and Benin
Source: Malar J. 2020 Aug 12;19:288. doi: 10.1186/s12936-020-03364-9 (PMC7424989; doi:10.1186/s12936-020-03364-9)
Supplement: Supplementary file 4 — Additional file 4. Liberia DTS study -DTS and RDT control Testing using new DTS. [file 12936_2020_3364_MOESM4_ESM.docx]

**Additional file 4**

**Liberia DTS study -DTS and RDT control Testing using new DTS**

**Result Legend**

Positive 1

Negative 2

Invalid 3

| **CDC New DTS (3D7) Testing** | | **Date: February 5, 2014** | | | | **Results** | **Band Intensity** | |
| --- | --- | --- | --- | --- | --- | --- | --- | --- |
|  |  |  | | **Parasite Density** |  |  |  |  |
| **First Response**  **(From Slipway)** | | | 500 p/µl | | Test A | 1 | 1+ |  |
|  |  |  | |  | Test B | 1 | 2+ |  |
|  |  |  | |  |  |  |  |  |
|  |  |  | | 1000 p/µl | Test A | 1 | 3+ |  |
|  |  |  | |  | Test B | 1 | 3+ |  |
|  |  |  | |  |  |  |  |  |
|  |  |  | |  |  |  |  |  |
|  |  |  | |  |  |  |  |  |
| **First Response (CDC)** | |  | | 500 p/µl | Test A | 1 | 3+ |  |
|  |  |  | |  | Test B | 1 | 2+ |  |
|  |  |  | |  |  |  |  |  |
|  |  |  | | 1000 p/µl | Test A | 1 | 4+ |  |
|  |  |  | |  | Test B | 1 | 4+ |  |
|  |  |  | |  |  |  |  |  |

| **First Response (From RL)** | |  | 500 p/µl | Test A | 1 | 2+ |
| --- | --- | --- | --- | --- | --- | --- |
|  |  |  |  | Test B | 1 | 2+ |
|  |  |  |  |  |  |  |
|  |  |  | 1000 p/µl | Test A | 1 | 4+ |
|  |  |  |  | Test B | 1 | 4+ |
|  |  |  |  |  |  |  |
|  |  |  |  |  |  |  |
|  |  |  |  |  |  |  |
| **First Response (CDC)** | |  | 500 p/µl | Test A | 1 | 3+ |
|  |  |  |  | Test B | 1 | 3+ |
|  |  |  |  |  |  |  |
|  |  |  | 1000 p/µl | Test A | 1 | 4+ |
|  |  |  |  | Test B | 1 | 4+ |
